# Clinical implications of augmented renal clearance after unrelated single cord blood transplantation in adults

**DOI:** 10.1007/s12185-023-03669-w

**Published:** 2023-10-18

**Authors:** Takaaki Konuma, Kosuke Takano, Maki Monna-Oiwa, Masamichi Isobe, Seiko Kato, Satoshi Takahashi, Yasuhito Nannya

**Affiliations:** 1 Department of Hematology/Oncology, The Institute of Medical Science, The University of Tokyo, 4-6-1, Shirokanedai, Minato-ku, Tokyo, 108-8639 Japan; 2 Division of Clinical Precision Research Platform, The Institute of Medical Science, The University of Tokyo, Tokyo, Japan

**Keywords:** Augmented renal clearance, Cord blood transplantation, Creatinine clearance, Mortality, Bloodstream infection

## Abstract

Augmented renal clearance (ARC) is a phenomenon characterized by increased renal functionality, which can impact the pharmacokinetics and pharmacodynamics of antimicrobial drugs eliminated by the kidneys. It is a potential concern for infection treatment. Cord blood transplantation (CBT) is primarily impeded by delayed neutrophil recovery and immune reconstitution, thereby increasing susceptibility to infection. However, the clinical implications of ARC following CBT remain unexplored. We retrospectively assessed the influence of ARC on post-transplant outcomes at various time points in 194 adult recipients of single-unit unrelated CBT between 2007 and 2022 at our institution. ARC was observed in 52.9% of patients at 1 day, 39.8% at 15 days, and 26.5% at 29 days post-CBT. ARC was not significantly associated with bloodstream infection, acute graft-versus-host disease, or veno-occlusive disease/sinusoidal obstruction syndrome at any time point. ARC at 1 day, 15 days, and 29 days post-CBT was not significantly associated  with overall survival, non-relapse mortality, or relapse rates. These findings suggest that ARC is common in adults during the early stages of CBT, but does not discernibly influence clinical outcomes or post-CBT complications.

## Introduction

Augmented renal clearance (ARC) is a phenomenon characterized by enhanced renal functionality frequently observed among critically ill individuals [1–3]. ARC possesses the potential to induce therapeutic inefficacy and reduce systemic drug exposure for renally cleared medications, thereby potentially compromising the efficacy of infection treatment [4–6]. Noteworthy risk factors for ARC include younger age, male gender, trauma, traumatic brain injury, hematological malignancies, and neutropenia [1, 2].

Cord blood transplantation (CBT) from unrelated donors represents an alternative approach for adult patients lacking human leukocyte antigen (HLA)-matched related or unrelated donors [7–9]. CBT is primarily hindered by delayed neutrophil recovery and immune reconstitution, heightening the risk of infectious complications [10–14]. Furthermore, since the glomerular filtration rate will ultimately determine the fate of the drug clearance, even for drugs that are first metabolized by the liver or intestine, such as chemotherapeutic drugs, immunosuppressants, and antifungal drugs, it is necessary to take into account the potential impact of ARC on the majority of drugs used in CBT [1]. Thus, the substantial effect of ARC on medication clearance might have consequences for post-transplant complications, including bloodstream infection (BSI), acute graft-versus-host disease (GVHD), and veno-occlusive disease/sinusoidal obstruction syndrome (VOD/SOS) after CBT. Nonetheless, the clinical implications of ARC after CBT remain unexplored. We postulated that ARC could influence outcomes following CBT and impact post-transplant complications. To investigate this, we conducted a retrospective analysis to assess whether ARC influences clinical outcomes and post-transplant complications in adults who underwent CBT.

## Methods

### Patients and CBT procedures

This retrospective study incorporated data from 194 adult patients who underwent single-unit unrelated CBT as their initial allogeneic hematopoietic cell transplantation (HCT) at our institution between March 2007 and December 2022. Unrelated cord blood units were procured from cord blood banks in Japan. Treating physicians determined the conditioning regimens and GVHD prophylaxis. Supportive care, including antibacterial, antifungal, and antiviral prophylaxis, as well as transfusion practices, were largely standardized across all patients [7, 14]. The Institutional Review Board of the Institute of Medical Science, University of Tokyo, granted approval for this retrospective study (2021–110-0331).

### Objectives and definitions

The primary objective of this retrospective study was to explore the influence of ARC at various time points on overall survival (OS), non-relapse mortality (NRM), and relapse rates following CBT. The secondary objectives were to examine the association between ARC and post-transplant complications, including BSI, acute GVHD, and VOD/SOS following CBT. BSI was defined as the isolation of bacteria from blood cultures between the day of CBT and 30 days after CBT. Confirmation of BSI caused by coagulase-negative *Staphylococcus* required the presence of two separate positive blood cultures [14]. The diagnosis of acute GVHD and VOD/SOS was based on previously established standard criteria.

Creatinine clearance (CrCl) was assessed through 24-h urine collection at least once a week during hospitalization for allogeneic HCT as a standardized procedure in our hospital. CrCl was calculated using the conventional formula: CrCl (mL/min) = urine volume (ml/min) × urinary creatinine (mg/dl)/serum creatinine (mg/dl), with adjustment for body surface area (BSA) determined by the Du Bois formula: corrected CrCl = CrCl × (1.73 m^2^/BSA). ARC was defined as a CrCl value of ≥ 130 mL/min/1.73m^2^ [2].

### Measurement of drug trough levels

The serum vancomycin (VCM) trough levels were measured at our hospital using two techniques, depending on the treatment period: the fluorescence polarization immunoassay (FPIA) (March 2007 to January 2012) and the chemiluminescent enzyme immunoassay (from February 2012), as previously described [15]. In a commercial laboratory (SRL, Tokyo, Japan), serum teicoplanin (TEIC) trough levels were tested using two techniques depending on the treatment period: the FPIA (from March 2007 to March 2013) and the latex agglutination turbidimetric immunoassay (from April 2013). Our hospital monitored serum cyclosporine (CSP) trough levels using two techniques, depending on the treatment period: the FPIA (from March 2007 to February 2012) and the chemiluminescent immunoassay (from March 2012).

### Statistical analysis

Group comparisons were conducted using Fisher's exact test for categorical variables. Continuous variables were compared using the Mann–Whitney U test. The probability of OS was estimated utilizing the Kaplan–Meier method, with differences assessed via the log-rank test. NRM and relapse probabilities were estimated using cumulative incidence curves to account for competing risks, and differences were evaluated using Gray's test. Regarding ARC at 15 and 29 days, the landmark days were set at 14 and 28 days after CBT, respectively, to evaluate the corresponding ARC values. Multivariate analysis was conducted employing a Cox proportional hazards model for overall mortality and a Fine and Gray model for NRM and relapse. The multivariate analysis included the following factors as covariates: ARC (yes vs. no), age (< 45 vs. ≥ 45 years), gender (male vs. female), HCT-Specific Comorbidity Index (< 3 vs. ≥ 3), refined disease risk index (low/intermediate vs. high/very high), cryopreserved cord blood total nucleated cell dose (< 2.5 × 10^7^/kg vs. ≥ 2.5 × 10^7^/kg), HLA disparities defined as high-resolution for HLA-A, -B, and -DR (< 3 vs. ≥ 3), and conditioning regimen (total body irradiation [TBI] ≥ 10 Gy-based vs. TBI < 10 Gy-based). GVHD prophylaxis (CSP plus methotrexate [MTX] vs. CSP plus mycophenolate mofetil) was not included in the variables of the multivariate analysis because selection for GVHD prophylaxis was associated with the type of conditioning regimen (*P* < 0.001 by Fisher's exact test). The significance level was set at *P* < 0.05, and all statistical analyses were performed using EZR software version 1.61 (Saitama Medical Center, Jichi Medical University, Saitama, Japan) [16] and GraphPad Prism 9 for Mac OS X (GraphPad Software Inc, San Diego, CA).

## Results

### Patient characteristics

Table 1 illustrates the characteristics of the patients enrolled in this study. The median age of patients at the time of CBT was 46.5 years. Acute myeloid leukemia accounted for the majority of cases, comprising 51% of the total. The predominant conditioning regimens employed were myeloablative regimens based on TBI with a dose of ≥ 10 Gy (78%), while CSP plus MTX were the most commonly utilized GVHD prophylaxis (78%).

**Table 1:** Patient characteristics

Characteristic	Value
Number of patients	194
Median age at CBT, (IQR) years	46.5 (37–56)
Sex	
Male	123 (63%)
Female	71 (37%)
HCT-CI	
0–2	164 (85%)
≥ 3	30 (15%)
Diagnosis	
AML	98 (51%)
ALL	39 (20%)
MDS	29 (15%)
CML	8 (4%)
NHL/ATL	7 (4%)
MPN/CMML	7 (4%)
Mastocytosis	1 (1%)
CAEBV/SAA	5 (3%)
Refined disease risk index	
Low/Intermediate and benign disorders	100 (52%)
High/very high	93 (48%)
Not available	1 (< 1%)
Cryopreserved cord blood TNC dose, (IQR) × 10^7^/kg	2.59 (2.17–3.25)
Cryopreserved cord blood CD34^+^ cell dose, (IQR) × 10^5^/kg	1.02 (0.78–1.26)
HLA disparities*	
0–2	89 (46%)
≥ 3	105 (54%)
Conditioning regimen	
TBI ≥ 10 Gy-based regimens	152 (78%)
TBI < 10 Gy-based regimens	42 (22%)
GVHD prophylaxis	
CSP with MTX	151 (78%)
CSP with MMF	43 (22%)

CBT, cord blood transplantation; IQR, interquartile range; HCT-CI, hematopoietic cell transplantation-specific comorbidity index; AML, acute myeloid leukemia; ALL, acute lymphoblastic leukemia; MDS, myelodysplastic syndrome; CML, chronic myelogenous leukemia; NHL, non-Hodgkin’s lymphoma; ATL, adult T-cell leukemia; MPN myeloproliferative neoplasm; CMML, chronic myelomonocytic leukemia; CAEBV, chronic active Epstein-Barr virus infection; SAA, severe aplastic anemia; TNC, total nucleated cell; HLA, human leukocyte antigen; TBI, total body irradiation; GVHD, graft-versus-host disease; CSP, cyclosporine; MTX, methotrexate; MMF, mycophenolate mofetil

*HLA disparities between cord blood graft and recipient were defined as a high-resolution for HLA-A, HLA-B, and HLA-DR

### CrCl values and ARC at each time point following CBT

The median CrCl values at various time points after CBT were as follows: 133.6 ml/min (Interquartile range [IQR], 104.4–165.8 ml/min) at 1 day, 118.0 ml/min (IQR, 88.6–147.6 ml/min) at 15 days, and 105.1 ml/min (IQR, 74.1–132.8 ml/min) at 29 days. ARC was observed in 100 (52.9%) of the 189 assessable patients at 1 day, 77 (39.8%) of the 193 assessable patients at 15 days, and 50 (26.5%) of the 188 assessable patients at 29 days after CBT.

Among adult patients who received allogeneic HCT from a matched sibling donor (n = 22), a matched unrelated donor (n = 18), or a haploidentical donor (n = 1) during the study period in our hospital, ARC was observed in 19 (48.7%) of the 39 assessable patients at 1 day, 13 (32.5%) of the 40 assessable patients at 15 days, and 9 (22.5%) of the 40 assessable patients at 29 days after HCT. The incidences of ARC at each time point were comparable between CBT and HCT from adult donors (Table 2).

**Table 2:** The incidences of ARC according to donor type

	CBT	HCT from adult donors	
Day1			0.725
No ARC	89 (47.1%)	20 (51.3%)	
ARC	100 (52.9%)	19 (48.7%)	
Day15			0.476
No ARC	116 (60.1%)	27 (67.5%)	
ARC	77 (39.8%)	13 (32.5%)	
Day29			0.693
No ARC	138 (73.4%)	31 (77.5%)	
ARC	50 (26.5%)	9 (22.5%)	

ARC, augmented renal clearance; CBT, cord blood transplantation; HCT, hematopoietic cell transplantation

### VCM, TEIC, and CSP trough levels according to ARC

VCM were administered, and VCM trough levels were evaluated within 4 days at 1 day (n = 92), 15 days (n = 136), and 29 days (n = 57) after CBT. TEIC was administered, and TEIC trough levels were evaluated within 4 days at 1 day (n = 12), 15 days (n = 32), and 29 days (n = 9) after CBT. CSP was administered, and CSP trough levels were evaluated within 4 days at 1 day (n = 183), 15 days (n = 170), and 29 days (n = 159) after CBT.

**Fig. 1:**
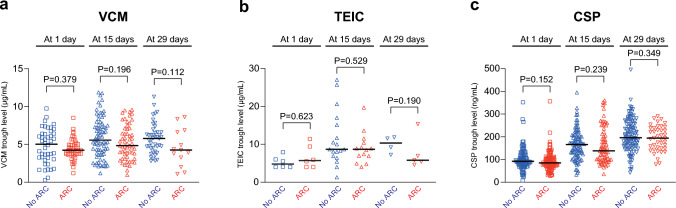
VCM (**a**), TEIC (**b**), and CSP (**c**) trough levels between augmented renal clearance (ARC) and no ARC groups at 1 day, 15 days, and 29 days after CBT. The lines represent the median value. Group comparisons were conducted using the Mann–Whitney U test

The patient group exhibiting ARC at 29 days displayed slightly lower VCM (P = 0.112) and TEIC (P = 0.190) trough levels compared to those without ARC, although these differences did not reach statistical significance (Fig. 1a, b). The patient group exhibiting ARC did not affect VCM and TEIC trough levels at 1 day or 15 days (Fig. 1a, b). There were no significant associations between ARC and CSP trough levels at each time point (Fig. 1c).

### Impact of ARC on BSI, acute GVHD, and VOD/SOS

The patient group exhibiting ARC at 1 day displayed a slightly higher proportion of BSI compared to those without ARC (19.0% vs. 10.1%, *P* = 0.102), although this difference did not reach statistical significance. Additionally, there were no significant associations between ARC at each time point and the occurrence of BSI, acute GVHD, or VOD/SOS (Table 3).

**Table 3:** Post-transplant complications according to ARC at each time point

	ARC at 1 day			ARC at 15 days			ARC at 29 days		
	No ARC	ARC	*P* value	No ARC	ARC	*P* value	No ARC	ARC	*P* value
BSI	9/89 (10.1%)	19/100 (19.0%)	0.102	17/116 (14.6%)	11/77 (14.2%)	1.000	20/138 (14.4%)	5/50 (10.0%)	0.478
Grade II–IV acute GVHD	64/84 (76.1%)	78/93 (83.8%)	0.257	88/109 (80.7%)	59/73 (80.8%)	1.000	109/135 (80.7%)	36/45 (80.0%)	1.000
Grade III–IV acute GVHD	13/83 (15.6%)	12/91 (13.1%)	0.671	19/107 (17.7%)	9/72 (12.5%)	0.405	22/134 (16.4%)	5/44 (11.3%)	0.479
VOD/SOS	4/89 (4.4%)	4/100 (4.0%)	1.000	7/116 (6.0%)	1/77 (1.2%)	0.148	5/138 (3.6%)	1/50 (2.0%)	1.000

BSI, bloodstream infection; GVHD, graft-versus-host disease; VOD/SOS, veno-occlusive disease/sinusoidal obstruction syndrome

Events / assessable patients (%) are shown

### Impact of ARC on OS, NRM, and relapse

**Fig. 2:**
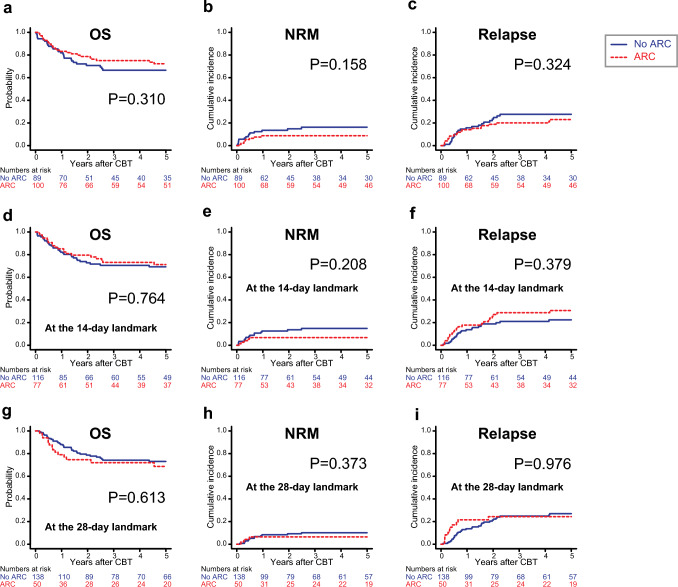
The impact of augmented renal clearance (ARC) on the overall survival (OS), non-relapse mortality (NRM), and relapse rate in adult patients who underwent single-unit cord blood transplantation (CBT). Kaplan–Meier survival curves were employed to depict OS, while cumulative incidence curves were used to represent NRM and relapse. These curves were plotted both without landmark (**a**–**c**) and with a conditional landmark analysis conducted at both 14 (**d**–**f**) and 28 days (**g**–**i**) after CBT. Group comparisons were conducted using the log-rank test for OS and Gray's test for NRM and relapse

Univariate analysis demonstrated that ARC at 1 day, 15 days, and 29 days post-CBT was not associated with the probability of OS or the cumulative incidences of NRM and relapse (Fig. 2). In the multivariate analysis, ARC following CBT at each time point was also not significantly associated with the probabilities of OS, NRM, or relapse rates (Table 4).

**Table 4:** Multivariable analysis of overall mortality, non-relapse mortality (NRM), and relapse rate

	Overall mortality		NRM		Relapse	
	HR (95% CI)	*P* value	HR (95% CI)	*P* value	HR (95% CI)	*P* value
Without landmark						
ARC at 1 day	0.86 (0.50–1.48)	0.603	1.07 (0.42–2.71)	0.880	0.63 (0.34–1.17)	0.150
Age ≥ 45 years	2.18 (1.15–4.14)	**0.016**	3.74 (1.15–12.15)	**0.028**	1.67 (0.83–3.35)	0.140
Female recipients	0.28 (0.14–0.57)	** < 0.001**	0.08 (0.01–0.38)	**0.001**	0.96 (0.48–1.91)	0.920
HCT-CI ≥ 3	1.21 (0.59–2.47)	0.586	1.97 (0.68–3.64)	0.210	0.89 (0.35–2.22)	0.810
rDRI high/very high	2.51 (1.42–4.43)	**0.001**	1.58 (0.68–3.64)	0.280	3.39 (1.80–6.37)	** < 0.001**
Cord blood TNC ≥ 2.5 × 10^7^ /kg	1.33 (0.76–2.33)	0.316	3.14 (1.31–7.50)	**0.010**	0.66 (0.37–1.18)	0.170
HLA disparities ≥ 3	0.84 (0.49–1.44)	0.541	1.09 (0.48–2.45)	0.820	0.67 (0.35–1.28)	0.230
TBI < 10 Gy-based regimens	1.26 (0.64–2.50)	0.493	2.58 (1.00–6.68)	0.050	0.41 (0.18–0.95)	**0.040**
Landmark at 14 days						
ARC at 15 days	0.95 (0.54–1.65)	0.857	0.99 (0.38–2.57)	0.990	1.17 (0.65–2.10)	0.600
Age ≥ 45 years	2.11 (1.11–4.00)	**0.021**	3.60 (1.08–12.00)	**0.037**	1.62 (0.81–3.23)	0.170
Female recipients	0.28 (0.14–0.58)	** < 0.001**	0.09 (0.02–0.38)	**0.001**	0.84 (0.43–1.67)	0.640
HCT-CI ≥ 3	1.26 (0.63–2.55)	0.504	1.98 (0.75–5.23)	0.170	1.08 (0.45–2.57)	0.850
rDRI high/very high	2.44 (1.37–4.34)	**0.002**	1.47 (0.62–3.43)	0.370	3.56 (1.87–6.77)	** < 0.001**
Cord blood TNC ≥ 2.5 × 10^7^ /kg	1.27 (0.72–2.25)	0.395	2.81 (1.17–6.74)	**0.021**	0.74 (0.42–1.31)	0.310
HLA disparities ≥ 3	0.88 (0.51–1.52)	0.659	1.21 (0.52–2.81)	0.640	0.63 (0.32–1.23)	0.180
TBI < 10 Gy-based regimens	1.25 (0.63–2.50)	0.512	2.41 (0.94–6.14)	0.064	0.48 (0.21–1.08)	0.078
Landmark at 28 days						
ARC at 29 days	1.22 (0.65–2.31)	0.528	0.84 (0.21–3.36)	0.810	1.05 (0.50–2.18)	0.890
Age ≥ 45 years	2.13 (1.10–4.10)	**0.023**	4.11 (1.08–15.60)	**0.037**	1.67 (0.83–3.36)	0.150
Female recipients	0.35 (0.17–0.72)	**0.004**	0.14 (0.03–0.63)	**0.011**	0.77 (0.39–1.53)	0.460
HCT-CI ≥ 3	1.22 (0.58–2.55)	0.584	1.95 (0.64–5.91)	0.230	1.09 (0.46–2.58)	0.830
rDRI high/very high	2.06 (1.14–3.71)	**0.015**	0.86 (0.34–2.21)	0.770	3.84 (2.02–7.32)	** < 0.001**
Cord blood TNC ≥ 2.5 × 10^7^ /kg	1.07 (0.59–1.93)	0.818	1.86 (0.74–4.68)	0.180	0.79 (0.44–1.42)	0.450
HLA disparities ≥ 3	0.91 (0.51–1.62)	0.762	1.28 (0.55–2.96)	0.560	0.61 (0.32–1.18)	0.150
TBI < 10 Gy-based regimens	1.08 (0.52–2.26)	0.819	2.05 (0.72–5.86)	0.180	0.51 (0.23–1.14)	0.100

ARC, augmented renal clearance; HCT-CI, hematopoietic cell transplantation-specific comorbidity index; rDRI, refined disease risk index; TNC, total nucleated cell; HLA, human leukocyte antigen, TBI, total body irradiation; HR, hazard ratio; CI, confidence interval

The *P *values in bold are statistically significant (< 0.05)

## Discussion

Our study demonstrated that ARC is frequently observed in adults during the early stages following CBT, with a prevalence ranging from 26.5 to 52.9%, comparable to that seen in intensive care unit (ICU) patients [2]. ARC has been shown to have an impact on the pharmacokinetics and pharmacodynamics of antimicrobial drugs eliminated by the kidneys, such as meropenem, piperacillin/tazobactam, and VCM, potentially leading to therapeutic failure in infection treatment [3–6]. In fact, consistent with a previous report indicating the influence of ARC on VCM clearance in children with febrile neutropenia after HCT [17], our previous study revealed a correlation between ARC and lower initial trough levels of VCM in recipients of CBT [15]. However, our current study revealed no influence of ARC on post-transplant complications or clinical outcomes in CBT recipients. While most previous studies have shown no impact of ARC on mortality in patients receiving certain antibiotics [18, 19], a Spanish group demonstrated lower ICU mortality in patients with ARC [20]. It is plausible that the presence of ARC indicates preserved renal function, potentially leading to improved clinical outcomes. Overall, these findings suggest that the prognostic implications of ARC in various clinical settings remain unclear.

Apart from antibiotics, it is conceivable that ARC might also contribute to therapeutic failure of chemotherapeutic drugs eliminated by the kidneys. However, the exact impact of ARC on the efficacy of such drugs remains uncertain. Thus, we hypothesized that ARC might be associated with an increased risk of relapse after CBT. Nonetheless, our findings revealed that ARC did not elevate the risk of relapse following CBT.

Our study could not demonstrate higher drug clearance among patients with ARC. This might be partly due to the adjustment of some drug dosages according to drug trough levels by treating physicians. These findings could contribute to the lack of clinical implications of ARC after CBT in adults.

In conclusion, our data underscore the common occurrence of ARC in adults during the early stages after CBT. However, we could not demonstrate higher drug clearance, such as VCM, TEIC, and CSP, among patients with ARC. ARC was not significantly associated with the development of BSI, acute GVHD, or VOD/SOS at any time point. Furthermore, ARC at each time point did not discernibly influence clinical outcomes, including OS, NRM, and relapse following CBT. Nevertheless, it is important to note that our study was a retrospective, single-institute analysis conducted in Japan, with a limited number of patients. Further research is warranted to elucidate the effects of ARC on clinical outcomes in the field of hematology and HCT.

## Data Availability

The data supporting this study's findings are available from the corresponding author upon reasonable request.
